# Cell Biology of Galectins: Novel Aspects and Emerging Challenges

**DOI:** 10.3390/biom12060744

**Published:** 2022-05-25

**Authors:** Alexander V. Timoshenko

**Affiliations:** Department of Biology, Western University, London, ON N6A 5B7, Canada; atimoshe@uwo.ca

Galectins are a family of soluble β-galactoside-binding proteins with diverse glycan-dependent and glycan-independent functions outside and inside the cell [1,2,3]. There are sixteen recognized mammalian galectin genes, and their expression profiles are very different between cell types, tissues, and species [4,5,6]. Galectins are known to be involved in regulating multiple processes in cells under normal, stress, and pathological conditions, which suggest they are potential candidates for biomedical applications. However, current success in this direction is challenging, mostly due to the complex network of interacting galectins in cells, different modes of their action, and association with diverse fundamental cellular functions (such as cell growth, differentiation, stemness, apoptosis, autophagy, phagocytosis, and cellular interactions). Functions of galectins depend on their localization in specific cellular compartments and organelles (cytosol, cytoskeleton, mitochondria, nucleus, lysosomes, and plasma membrane) and related intracellular trafficking and secretion, which occurs through non-classical pathways [7,8]. An integrated vision of the galectin cell biology is still warranted, and advanced studies of galectin post-translational modifications, the transcriptional regulation of galectin gene expression, and galectin-mediated transmembrane signaling are highly regarded. This Special Issue covers recent progress in the field of cell biology of galectins, relevant concepts of galectin regulatory mechanisms, and biomedical aspects of these unique multifunctional proteins.

Five articles in this Special Issue represent original and novel research studies with both well-known galectin family members (galectins-1, -3, -4, and -9) and relatively poorly characterized galectins (rat galectin-5 and human galectin-16). Gatie et al. [9] explore the role of galectins in a model of extraembryonic endoderm differentiation and provide experimental evidence in support of the new concept of the *O*-GlcNAc-mediated regulation of galectin expression and secretion [10,11]. The findings from this study together with an excellent recent report from Hanover group [12] indicate that the secretion of galectin-3 is an *O*-GlcNAc-dependent process, which represents a new mechanism of unconventional secretion, requiring deglycosylation of released molecules. Although bioinformatics analysis suggests that all human galectin molecules have potential sites for *O*-GlcNAcylation [11], whether this mechanism works for other galectins remains to be investigated. Ayechu-Muruzabal et al. [13] demonstrate that secretion of galectin-3, -4, and -9 can be stimulated by specific types of galacto-oligosaccharides and CpG oligodeoxynucleotides in a complex transwell co-culture model of intestinal epithelial cells (human colon adenocarcinoma HT-29 cell line) with primary peripheral blood mononuclear cells, which ultimately supports the role of galectins in the mucosal immune response. In this context, the authors highlight the key immunomodulatory contribution of β-3′galactosyllactose, a component of human milk [14], which reveals an interesting aspect of health benefits associated with secreted galectins. In comparison, Jofre et al. [15] analyze the role of galectin-1 in mechanisms, allowing Gram-negative bacteria *Yersinia enterocolytica,* which causes gastrointestinal infections, to escape immunosurveillance and demonstrate the critical role of carbohydrate-dependent interaction with *Yersinia* outer proteins in this context. As such, the authors show that galectin-1 can bind to and protect the bacterial proteins from trypsin digestion and also contribute to the decrease in nitric oxide production by infected macrophages. Thus, the multifunctional role of galectins remains a challenging topic for health and disease control. The knowledge of tissue-specific expression of several galectins, such as galectin-12 in adipocytes and leukocytes [16] and galectin-16 in placenta [17], raises further insights into an inherent galectin-mediated regulation of cellular responses, including cellular differentiation. Currently, galectin-16 is at a very early stage of its elaboration and the article in this Special Issue introduces a model of trophoblastic differentiation of BeWo and JEG-3 cells associated with the upregulation of *LGALS16* gene expression [18]. In addition, bioinformatics analyses highlight possible transcriptional and post-transcriptional regulation of *LGALS16* as well as its associations with other tissues and human diseases. To wrap up the experimental part of this Special Issue, the article by Ruiz et al. [19] reports a comprehensive crystallographic characterization and ligand-binding specificity of rat galectin-5, a lectin with a unique N-terminal extension that is likely serving as a molecular binding switch.

Five review articles provide a comprehensive summary of the cell biology of galectins, as well as their functions and applications in normal and pathophysiological context. A review article by Thijssen [20] elegantly describes all the studied functions of endothelial galectins (galectin-1, -3, -8, and -9), making it easy for readers to understand the versatile functions of individual galectins in endothelial cell biology and angiogenesis. The role of commonly expressed galectin-3 is addressed in two reviews highlighting several important themes: (1) the diagnostic and therapeutic potential in cardiovascular diseases by Sygitowicz et al. [21] and (2) the complexity and controversy of galectin-3/pectin interactions in the context of dietary interference by Pedrosa et al. [22]. Finally, two reviews focus on galectin-7, an epithelial cell galectin with pro-apoptotic and pro-carcinogenic properties. The review by St-Pierre [23] provides exceptional information and clarifications on the complex relationship between galectin-7, p53, and MMP-9 molecules, which helps to clarify the role of galectin-7 in cancer cells. Sewgobind et al. [24] summarize the pathophysiological role of galectin-7 in multiple human diseases, justify a rationale to design small-molecule inhibitors of galectin-7, and present structure and examples of novel carbohydrate and non-carbohydrate compounds to be tested against cellular disorders including cancer.

To conclude, the collection of research and review articles in this Special Issue addresses novel and challenging aspects of cell biology of galectins that might catalyze further experimental investigations into the complex network of galectin molecules in cells.
